# What do we know about maternal guilt? A narrative review of its measurement, sources, and correlates

**DOI:** 10.3389/fsoc.2026.1963417

**Published:** 2026-10-30

**Authors:** Maria Stoicescu, Natalia Soboleva, Nevena Kulic, Giulia Dotti Sani

**Affiliations:** 1 Department of Political and Social Sciences, University of Pavia, Pavia, Italy; 2 Department of Social and Political Sciences, Universita degli Studi di Milano, Milan, Italy

**Keywords:** identity conflict, maternal guilt, motherhood, narrative review, social emotions

## Abstract

Maternal guilt is a complex social emotion shaped by individual circumstances, interpersonal relationships, and broader cultural and institutional expectations. This narrative review synthesizes 52 studies to examine the theoretical underpinnings of maternal guilt, its sources and its correlates. It adopts an interdisciplinary approach with a particular emphasis on sociological perspectives. Drawing on dramaturgical theory and symbolic interactionism, the review conceptualizes maternal guilt as arising from tensions between lived experiences of motherhood, socially established feeling rules, competing identities, and cultural ideals of the “good mother.” Four dominant sources of maternal guilt are identified: maternal responsibility, identity conflict, interactions with children, and the idealization of motherhood. The review further highlights the multilevel factors associated with maternal guilt, including individual characteristics and social beliefs, family circumstances, domestic support, financial conditions, workplace environments, and broader societal institutions and norms. The review identifies several gaps in the literature, including the limited availability of cross-national comparisons, insufficient attention to socioeconomically and socially diverse groups of mothers, and the lack of systematic comparisons between maternal and paternal guilt. Moreover, although existing research has made important contributions through diverse quantitative, psychometric, and qualitative approaches, methodological heterogeneity limits the comparability and generalizability of findings across studies and contexts. Emerging research areas include the influence of digital technologies and social media on maternal guilt, variations across children’s developmental stages, and the role of anticipated guilt among women who have not yet become mothers. Finally, the review calls for more longitudinal, experimental, and comparative research to clarify how maternal guilt develops over time and how social policies and organizational practices may shape its experience. By situating maternal guilt within its broader social and structural context, this review advances a sociological understanding of motherhood and identifies priorities for future research.

## Introduction

One challenge of motherhood lies in the gap between personal expectations and societal judgment, a tension that often manifests as maternal guilt: the feeling of failing to live up to cultural ideals of the “good mother,” who is expected to provide near-total physical, psychological, and intellectual dedication to children. These standards stem from the caregiving role traditionally assigned to women, rooted in essentialist beliefs that women are naturally suited to be primary caregivers. This essentialism underlies what Hays (1996) calls the ideology of “intensive mothering,” which positions mothers as fundamentally different from fathers and irreplaceably central to their children’s wellbeing. Women who internalize this belief may come to see any time away from their children, including time spent working, as denying them the best possible care and experience this as a kind of moral failure. The idealization of motherhood has been also referred to as the “myth of motherhood” (Rich, 2021), underlining that motherhood is often treated as an instinctual and fulfilling role rather than as a socially constructed ideal.

The impossibility of conforming to motherhood standards can lead to feelings of maternal guilt (Sutherland, 2010), an emotional experience that has been linked to lower life satisfaction (Foucreault et al., 2023), depression (Derella and Milan, 2021), and poorer mental health (Kim et al., 2023; Siddiqi et al., 2025). Maternal guilt also results from the perceived incompatibility between the “ideal worker” requirements (Acker, 1990) and “ideal mother” expectations (Hays, 1996). Many mothers adjust their working lives in an attempt to ease feelings of guilt about not spending enough time with their children. Some reduce their working hours, while others step away from the labor market altogether so they can devote more time to family life (Rubin and Wooten, 2007; Aarntzen et al., 2019; Calarco et al., 2021). Yet, cutting back on work does not always mean that mothers spend more time with their children or experience greater well-being (Aarntzen et al., 2019). Maternal guilt can arise from many different sources, even in the absence of paid work. Overall, this evidence suggests that maternal guilt can significantly undermine mothers’ mental health and overall well-being, making it a central factor to examine in efforts to promote better motherhood experiences.

In this article, we argue that a deeper understanding of maternal guilt is essential to identify the factors that contribute to it and fully grasp its impact on women. Previous reviews have examined guilt and shame in relation to workplace and household contexts (Sutherland, 2010), gender differences in work–family guilt (Korabik, 2015), maternal guilt in relation to societal motherhood ideals (Constantinou et al., 2021), and guilt associated with infant care (Benoit et al., 2016; Jackson et al., 2021, 2022). Building on this work, the present review provides a comprehensive overview of maternal guilt by discussing its theoretical background, reviewing its measurement, proposing a classification of its sources, examining its key correlates and cross-national variation, and identifying priorities for future research. The review focuses primarily on guilt, understood as the feeling that one has failed to fulfil a moral obligation through one’s own behavior, rather than on shame, which arises from the perceived failure to meet broader societal standards (Orth et al., 2006). Nevertheless, the literature reviewed occasionally addresses shame alongside guilt, reflecting the conceptual overlap between these two emotions.

Our study draws on a broad selection of studies that have explored maternal guilt using both quantitative and qualitative approaches. It covers articles published in English from March 1999 up to July 2026. Searches, initiated in March 2024, were conducted in Scopus, Web of Science Core Collection, and Google Scholar using the terms “maternal guilt,” “mother guilt,” “parental guilt,” and “motherhood and guilt.” Articles were included if they measured or analyzed maternal or parental guilt and included mothers as participants. The same screening procedure—title and abstract review followed by full-text assessment—was applied to both quantitative and qualitative studies.

As a result of this process, the review includes 52 studies: 23 use quantitative methods, 28 use qualitative methods, and one uses mixed methods. The studies draw on data from 20 countries worldwide: Australia, Bulgaria, Canada, China, Finland, Germany, Hungary, Iceland, India, Ireland, Israel, Italy, the Netherlands, Portugal, South Korea, Spain, Sweden, Turkey, UK, and the US. By including such a wide range of empirical studies conducted across various regions of the world and based on different research methods, our study delivers a comprehensive investigation capturing the complexity and diversity of maternal guilt.

The review begins by presenting two theoretical frameworks through which maternal guilt can be understood from a sociological perspective. It then examines how maternal guilt is measured in quantitative and qualitative research and classifies dominant sources of guilt based on recurring patterns in the literature. Next, it reviews the key individual, situational, and societal correlates of maternal guilt, and then examines the available, albeit limited, cross-national evidence. The final sections discuss the contribution of the article and outline directions for future research.

## Theoretical explanations for the emergence of maternal guilt

Maternal guilt can be understood as a socially constructed emotion rather than merely an individual psychological experience. Sociological perspectives such as dramaturgical theory and symbolic interactionism (Turner and Stets, 2006) help explain how cultural expectations, social relationships, and tensions between different identities shape mothers’ experiences of guilt. This section draws on these complementary theoretical perspectives to examine maternal guilt.

From a dramaturgical or impression management perspective, women have historically been expected to prioritize motherhood and derive deep fulfilment from it. However, as women’s roles and aspirations have expanded beyond the domestic sphere in modern societies, complete devotion to motherhood can become restrictive and emotionally demanding. Many mothers therefore experience a tension between how they are expected to feel and how they actually feel, often leading to feelings of guilt. This emotional conflict can be understood through Arlie Hochschild’s theory of feeling rules and emotion work (Hochschild, 1979). Feeling rules refer to the socially prescribed emotions considered appropriate in particular situations and roles, while emotion work describes the effort individuals make to align their emotions with these expectations. For mothers, feeling rules may include assumptions such as: a mother should always enjoy spending time with her child, or motherhood should feel naturally fulfilling and rewarding. These emotional expectations are difficult to sustain in everyday modern life. Research shows that mothers frequently experience negative emotions such as frustration, exhaustion, ambivalence, or resentment, and these feelings may intensify guilt because mothers often believe they are not supposed to feel this way (Rotkirch and Janhunen, 2010).

According to symbolic interactionist theories, emotions emerge through social interaction and the ongoing (also imagined) evaluation of the self by others. Individuals experience positive emotions when they believe their identities are validated, whereas negative emotions arise when those identities appear to be disapproved of or fail to meet social expectations (Turner and Stets, 2006). Building on Susan Shott’s work, maternal guilt can be understood as the emotional consequence of perceiving oneself as falling short of socially constructed ideals of motherhood. Shott (1979) conceptualizes guilt as a “role-taking” emotion, meaning that it arises when individuals believe they have harmed or disappointed someone with whom they share a meaningful social relationship. This highlights the fundamentally interactional nature of maternal guilt: mothers evaluate themselves through imagined or anticipated judgments from others and from internalized cultural norms surrounding “good motherhood.” Maternal guilt may therefore emerge not only when mothers feel insufficiently devoted to caregiving or perceive themselves as “bad mothers,” but also when they invest in alternative identities—such as professional, personal, or autonomous selves—that appear to compete with the maternal role. In this sense, maternal guilt reflects the tension between multiple identities and the persistent social expectation that motherhood should remain primary.

Overall, sociological theories show that maternal guilt is a socially shaped emotion rooted in cultural expectations surrounding motherhood. Cultural expectations about how mothers should feel, reinforced through everyday interactions, pressure mothers to meet idealized standards of caregiving and self-sacrifice. As a result, maternal guilt emerges when mothers perceive a gap between these social expectations and their own emotions, behaviors, or competing identities. The following section describes the approaches used to measure maternal guilt, an essential process that helps in understanding the extent of this phenomenon.

## Measuring maternal guilt

This section provides an overview of the quantitative and qualitative methods and instruments used to study maternal guilt, with selective discussion of study populations, research designs, and sampling strategies.

### Research relying on quantitative methods

In most of the quantitative empirical studies reviewed in this article, maternal (or parental) guilt is measured using self-report psychometric scales on samples of various types. Such scales employ sets of representative statements (i.e., items) aimed at capturing the feelings of guilt experienced by parents. Participants are typically asked to evaluate how frequently they experience guilt in various parenting situations. Responses are usually recorded on Likert-type scales, allowing researchers to quantify variations in guilt across individuals and circumstances (Table 1).

**Table 1:** Research relying on quantitative methods.

No.	Author(s)	Country	Sample size	Children’s age	Measurement of guilt
1	Martínez et al. (2011)	Spain	251 employed parents: 131 mothers and 120 fathers	Ages 3 to 6	Scale of Feelings of Guilt about Parenting
2	Liss et al. (2013)	US	181 mothers	Under 5 years	State Shame and Guilt Scale (SSGS; Marschall et al., 1994)
3	Borelli et al. (2016)	US	255 employed parents: 140 mothers and 115 fathers	Ages 1 to 3	Pomona Work and Family Assessment (PWFA narrative coding) and 6-item Positive and Negative Affect Schedule – Expanded Form (PANAS-X) guilt subscale
4	Borelli et al. (2017)	US	245 employed parents: 128 mothers and 117 fathers	Ages 1 to 3	Work-Interfering-With-Family Guilt Scale (WIFGS)
5	Dunford and Granger (2017)	UK	183 mothers	4 weeks to 1 year of age	Event-Related Shame and Guilt Scales (ERSGS; Orth et al., 2006) and Test of Self-Conscious Affect–3 (TOSCA-3)
6	Gonçalves et al. (2018)	Portugal	213 employed parents: 104 mothers and 109 fathers	Dependent children	Work-Family Guilt Scale (WFGS; McElwain, 2008)
7	Uysal Irak et al. (2020)	Türkiye	201 working mothers	Under 10 years	Employment-Related Guilt Scale (Aycan and Eskin, 2005)
8	Slobodin et al. (2020)	Israel	215 mothers	Ages 3 to 8	State Shame and Guilt Scale (SSGS; Marschall et al., 1994) guilt subscale
9	Miller and Strachan (2020)	Canada	143 mothers	Under 5 years	State Shame and Guilt Scale (SSGS; Marschall et al., 1994) guilt subscale
10	Aarntzen et al. (2021)	Bulgaria, Finland, Germany, Hungary, Netherlands, Portugal, Spain, Sweden, UK	2,601 employed parents (47.1% mothers)	Mean age 10.42 years	Single-item 5-point Likert scale on WFC guilt frequency
11	Maclean et al. (2021)	Australia	1,375 working mothers	Under 13 years	Work-Interfering-with-Family Guilt Scale (WIFGS; Borelli et al., 2017)
12	Derella and Milan (2021)	US	212 mothers	Ages 3 to 18	Open-ended Parent Development Interview (PDI-R) prompts analyzed via Linguistic Inquiry and Word Count (LIWC)
13	Kerr et al. (2021)	US	145 mothers	Aged 18 to 27 months	Single-item momentary guilt rating using an ecological momentary assessment design (EMA; 1–5 scale)
14	Law et al. (2021)	UK and Ireland	395 mothers	Aged 2 to 11 months	Harder Personal Feelings Questionnaire (PFQ-2) guilt subscale
15	Aarntzen et al. (2023)	Netherlands	251 employed parents (53.8% mothers)	Study 1: Mean 8 years; Study 2: under 13 years	Study 1: Imaginary WFC scenario rating; Study 2: 8-day daily diary WFC guilt
16	Wolfers et al. (2023)	Germany	158 mothers	Aged 0 to 6 years	Experience Sampling Study (situational smartphone guilt)
17	Foucreault et al. (2023)	Canada	289 employed parents (81.7% mothers)	Aged 5 to 10	Work-Family Guilt Scale (WFGS; McElwain, 2008)
18	Kim et al. (2023)	South Korea	419 single mothers	Not reported	Parenting Guilt Scale (7-item adaptation of Maternal Guilt Scale; Mann and Thornburg, 1987)
19	Song et al. (2025)	China	476 working mothers	Adolescents (exact age not reported)	10-item Chinese version of Guilt About Parenting Scale (GAPS; Haslam et al., 2020)
20	Siddiqi et al. (2025)	India and US	307 working mothers	Under 5 years	15-item Maternal Employment Guilt Scale (MEGS; Borelli et al., 2017)
21	Coyne et al. (2025)	US	474 US mothers	Ages 6 to 8	10-item Parental Media Guilt Scale (adapted from GAPS; Haslam et al., 2020)
22	Bayyar et al. (2026)	Turkey	401 working mothers	Ages 0 to 12	15-item Maternal Employment Guilt Scale (MEGS; Borelli et al., 2017; 14 items analyzed)
23	Kenny and Robinson (2026)	UK and Ireland	94 biological mothers	At least one child under 10 years	10-item Guilt About Parenting Scale (GAPS; Haslam et al., 2020) and 2-item State Shame and Guilt Scale (SSGS; Marschall et al., 1994) guilt subscale

Some scales are specifically designed to capture the guilt arising from the conflict between professional and family roles, reflecting a distinct dimension of maternal guilt. For example, the *Work-Interfering-with-Family Guilt Scale* (WIFGS) developed by Borelli et al. (2017) measures guilt about the perceived negative impact of work on family life among parents of toddlers in the US. This scale was also used to measure guilt among Australian working mothers with children up to 13 years of age (Maclean et al., 2021). It was also adapted into the *Maternal Employment Guilt Scale* (MEGS) by Yüce-Selvi and Kantaş (2019) to compare American and Indian working mothers (Siddiqi et al., 2025) and to examine employment guilt among Turkish working mothers (Bayyar et al., 2026). The *Work–Family Guilt Scale* (WFGS) developed by McElwain (2008), assesses guilt both work-interfering-with-family (WIFG) and family-interfering-with-work (FIWG). This scale was further used by Foucreault et al. (2023) in Canada and adapted to measure parental guilt among the Portuguese population (Gonçalves et al., 2018). In addition, Aycan and Eskin’s (2005) *Employment-Related Guilt Scale*, which measures guilt related to maternal employment, was used to investigate employment-related guilt among Turkish mothers (Uysal Irak et al., 2020). Collectively, these instruments conceptualize guilt as a consequence of work–family conflict.

Few instruments have been developed to assess maternal guilt as a multidimensional construct. One example is the *Maternal Guilt Scale*, originally developed by Mann and Thornburg (1987) used to measure guilt associated with the maternal role. More recently, Kim et al. (2023) adapted this scale for use in their study of South Korean single mothers. Instead, broader parenting measures have been more widely adopted in maternal guilt research. Among these, the *Guilt about Parenting Scale* (GAPS), developed by Haslam et al. (2020), has emerged as one of the most frequently used instruments for assessing the emotional aspects of parenting guilt, largely because of its adaptability across different contexts. Since its development, the GAPS has been adapted to assess maternal guilt among Chinese mothers (Song et al., 2025), maternal guilt related to children’s media use (Coyne et al., 2025), and maternal guilt among biological mothers in the United Kingdom and Ireland (Kenny and Robinson, 2026).

Other scales were not developed specifically to assess maternal or parental guilt but have been applied in maternal guilt research. For example, the *State Shame and Guilt Scale* (SSGS), originally developed by Marschall et al. (1994) has enabled researchers to examine associations between maternal guilt and related processes, as well as to distinguish guilt from shame (Liss et al., 2013; Miller and Strachan, 2020; Slobodin et al., 2020).

Beyond psychometric self-report scales, maternal guilt has also been assessed using a range of alternative methodologies. Some quantitative studies have assessed maternal guilt using *narrative and linguistic coding* (Borelli et al., 2016; Derella and Milan, 2021). In these studies, participants provide narrative responses that are then coded for expressions of guilt, either by blind raters (Borelli et al., 2016) or through automated linguistic analysis software (Derella and Milan, 2021). Other researchers have used *ecological momentary assessment* via smartphone surveys, in which mothers report their guilt in real time multiple times per day (Kerr et al., 2021). Maternal guilt has also been examined using broader measures of emotional functioning, such as the *Test of Self-Conscious Affect*, which assesses likely emotional responses to hypothetical transgressions (Dunford and Granger, 2017), and the *Harder Personal Feelings Questionnaire*, which measures the frequency of emotions including regret and mild guilt (Law et al., 2021). Finally, in some studies, guilt is measured via single-item, close-ended questions, such as “How often does it happen that you feel guilty about how your obligations at work are affecting your family?” (Aarntzen et al., 2021, p. 126). Although brief, these measures can efficiently capture specific aspects of maternal guilt.

Several of the reviewed quantitative studies examined parental guilt more broadly, enabling comparisons between mothers and fathers (Martínez et al., 2011; Borelli et al., 2016, 2017; Gonçalves et al., 2018; Aarntzen et al., 2019, 2021, 2023; Foucreault et al., 2023). In this literature, findings regarding gender differences are mixed. For instance, regarding work–family guilt, Gonçalves et al. (2018) found no significant differences between Portuguese mothers and fathers in either work-interference-with-family guilt or family-interference-with-work guilt. Instead, Aarntzen et al. (2023) found that mothers reported greater guilt than fathers when work interfered with family responsibilities. In most of the reviewed studies, however, mothers are overrepresented. A total of 16 quantitative studies focused exclusively on mothers. Among these, several studies have focused specifically on sub-groups, such as single mothers (Kim et al., 2023), student mothers (Maclean et al., 2021), public vs. private sector employed mothers (Bayyar et al., 2026), and full-time working mothers (Foucreault et al., 2023).

Regarding the temporal design of the included studies, most adopted a cross-sectional approach. However, a smaller number employed longitudinal or intensive repeated-measures designs (e.g., daily diaries and experience sampling) to track mothers’ experiences over time. Yet, most of these studies examined relatively short time frames rather than longer-term trajectories. For instance, Derella and Milan (2021) employed a short-term longitudinal design in which mothers were assessed at two time points over 1 month, whereas Song et al. (2025) conducted three survey waves separated by one-month intervals. Several studies implemented diary and experience sampling methods, assessing participants multiple times per day or daily over a week or longer to capture real-time fluctuations in maternal experiences (Aarntzen et al., 2019, 2023; Kerr et al., 2021; Wolfers et al., 2023).

An important factor distinguishing the design of quantitative studies on maternal guilt is the stage of parenthood, typically defined by the age of the children. This is a key consideration in the application of maternal guilt measures because caregiving demands, developmental challenges, and societal expectations vary across child developmental stages, shaping both the experience and expression of guilt. Accordingly, most studies have focused on mothers of infants, toddlers, and preschoolers, reflecting the particular demands of early childhood (Martínez et al., 2011; Liss et al., 2013; Borelli et al., 2016, 2017; Miller and Strachan, 2020; Slobodin et al., 2020; Kerr et al., 2021; Law et al., 2021; Wolfers et al., 2023). In contrast, fewer studies have focused on mothers of school-aged children (Uysal Irak et al., 2020; Aarntzen et al., 2021; Maclean et al., 2021; Coyne et al., 2025; Bayyar et al., 2026; Kenny and Robinson, 2026). Mothers of adolescents were the least targeted in quantitative studies of maternal guilt (Derella and Milan, 2021; Song et al., 2025).

Regarding sample characteristics, non-representative surveys were the most used tool to analyze maternal guilt. The recruitment of participants includes both offline methods, such as the use of posters, flyers and snowballing-based sampling to access participants (Miller and Strachan, 2020; Wolfers et al., 2023), as well as online recruitment such as social media posts, online groups announcements (Slobodin et al., 2020; Kerr et al., 2021; Maclean et al., 2021) and online advertisements on parenting websites (Dunford and Granger, 2017). Other modalities employed were field-based recruitment and accessing participants through Amazon’s Mechanical Turk (Derella and Milan, 2021), and email communication containing a link to an online questionnaire (Foucreault et al., 2023).

### Research relying on qualitative methods

In qualitative studies, the focus is primarily on mothers’ experiences of guilt, with comparatively limited attention given to fathers or the experience of parental guilt within couples. Across these studies, several recurring themes emerge, including mothers’ negotiations of work–family balance (Hillier, 2020; Collins, 2021; Manna et al., 2021; Öke Karakaya et al., 2021; Batram-Zantvoort et al., 2022), infant feeding, food preparation and the gendered labor of “foodwork” (Williams et al., 2013; Pescud and Pettigrew, 2014; Holcomb, 2017; Fielding-Singh and Cooper, 2023), the ways in which racial, ethnic, and migration-related contexts intensify and shape maternal guilt (Li, 2022, 2023; Montano et al., 2023; Miller-Roenigk et al., 2026), life-course transitions and the challenges of becoming and being a parent (Dudley-Marling, 2001; Baum, 2007; McDonald et al., 2007; Murray and Finn, 2012), and the processes through which maternal guilt is experienced, internalized, and reproduced in everyday life (Elvin-Nowak, 1999; Jackson and Mannix, 2004; Rotkirch and Janhunen, 2010) (Table 2).

**Table 2:** Research relying on qualitative methods.

No.	Author(s)	Country	Sample size	Children’s age	Measurement of guilt
1	Elvin-Nowak (1999)	Sweden	13 working mothers	Under 12 years	Phenomenological unstructured interviews and written case descriptions
2	Dudley-Marling (2001)	US	6 single mothers, 10 mothers from two-parent families and 7 couples	Aged 8 to 17 years	23 semi-structured interviews with parents of struggling learners
3	Seagram and Daniluk (2002)	Canada	8 mothers	Between 2 and 12 years	In-depth narrative/phenomenological interviews
4	Jackson and Mannix (2004)	Australia	20 mothers	Ages 15 to 31 years	Conversational-style feminist narrative interviews
5	Guendouzi (2006)	UK	8 female teachers (7 mothers)	Not reported	Discourse analysis of 42 audio-taped staffroom conversations over 7 months
6	Baum (2007)	Israel	2 mothers	Grown-up and dependent children	Qualitative clinical case studies (divorce initiators)
7	Rubin and Wooten (2007)	US	10 stay-at-home mothers	Aged 4 months to 11 years	In-depth, open-ended narrative interviews with highly educated mothers
8	McDonald et al. (2007)	Australia	6 mothers	Aged 12 to 21 years	Conversational-style in-depth interviews (parents of self-harming adolescents)
9	Rotkirch and Janhunen (2010)	Finland	63 mothers	Under 7 years	Qualitative content analysis of self-solicited written essays
10	Murray and Finn (2012)	UK	6 new mothers	Newborn infants	Semi-structured in-depth interviews on intrusive thoughts
11	Williams et al. (2012)	Australia, UK, US	28 education materials on parenting	Not applicable	Critical discourse analysis of parenting guidance materials (2000–2008)
12	Williams et al. (2013)	Australia	35 mothers	Under 2 years of age	Discourse analysis of 9 focus groups
13	Pescud and Pettigrew (2014)	Australia	35 mothers and 2 fathers (low-income)	Aged 5 to 9 years	6-month longitudinal study: self-introspections, individual interviews, and focus groups
14	Sullivan (2015)	UK	8 magazine articles	Not applicable	Media discourse analysis of work-life balance representations
15	Holcomb (2017)	US	22 first-time mothers	Newborn infants	Longitudinal study: 96 semi-structured interviews repeated at various time intervals
16	Hillier (2020)	Canada	11 mothers (grad students and faculty)	Not reported	Narrative inquiry: semi-structured interviews and 2 focus groups
17	Collins (2021)	Sweden, Germany, Italy, US	4 focal working mothers (135 in full study)	Under 5 years	Qualitative comparative case study based on cross-national in-depth interviews
18	Calarco et al. (2021)	US	55 mothers in prepandemic dual-earner couples	Under 2 years	In-depth interviews during COVID-19
19	Manna et al. (2021)	Italy	30 mothers who voluntarily resigned	Under 1 year at resignation	Grounded theory in-depth interviews 5 years post-resignation
20	Öke Karakaya et al. (2021)	Türkiye	17 working nurses	Not reported	Phenomenological face-to-face semi-structured interviews
21	Batram-Zantvoort et al. (2022)	Germany	17 working mothers	Dependent children	Qualitative email interviews across 3 waves during COVID-19 lockdown
22	Fielding-Singh and Cooper (2023)	US	35 college-educated mothers	Aged 12 to 19 years	In-depth semi-structured interviews on maternal foodwork and emotional work
23	Li (2022)	China	24 rural-to-urban migrant mothers	Dependent children	Grounded theory in-depth semi-structured interviews
24	Li (2023)	China	24 rural-to-urban migrant mothers	Dependent children	Grounded theory in-depth semi-structured interviews
25	Rúdólfsdóttir and Auðardóttir (2024)	Iceland	374 mothers and 76 fathers	Ages 0 to 17 years	Affective-discursive analysis of 450 qualitative online questionnaires
26	Montano et al. (2023)	US	12 working Latina mothers	Dependent children	Grounded theory semi-structured interviews
27	Karademir Hazır (2024)	UK	12 middle-class families	Under 4 years	Longitudinal qualitative study: 3 interview sessions over 6–8 months
28	Miller-Roenigk et al. (2026)	US	37 Black cisgender mothers	Not reported	Qualitative semi-structured interviews and thematic analysis on breastfeeding

Most of these studies assessed maternal guilt through interviews as the primary data collection method. Semi-structured (Collins, 2021; Montano et al., 2023; Miller-Roenigk et al., 2026) and in-depth interviews (Rubin and Wooten, 2007; Li, 2022, 2023; Fielding-Singh and Cooper, 2023) were the most used forms of individual interviewing. Several studies also employed unstructured or conversational interviews to encourage participants to engage in more spontaneous and free-flowing storytelling (Seagram and Daniluk, 2002; Jackson and Mannix, 2004; McDonald et al., 2007). Focus groups were used less frequently, as a way to explore collective sense-making and the ways in which mothers interacted with and compared their experiences to those of other mothers (Williams et al., 2013; Pescud and Pettigrew, 2014; Hillier, 2020).

A smaller number of studies adopted alternative qualitative approaches. Sullivan (2015), for example, analyzed eight articles published over 1 year in popular UK women’s magazines to examine representations of “Bad Mum Guilt.” Williams et al. (2012) analyzed 28 parenting manuals, pamphlets, and government documents to investigate how maternal feeding choices and the associated emotions are portrayed. Guendouzi (2006) examined 42 naturally occurring conversations among female teachers in a staff lounge over a six-month period to capture everyday discussions of maternal guilt. Two authors performed ethnographic research in addition to interviewing to gain depth into the maternal guilt expressions by observing and participating in daily tasks with participants (Li, 2022, 2023; Karademir Hazır, 2024).

Some researchers relied on open-ended questions, using content analysis to examine participants’ responses. For example, a large-scale Icelandic study collected 450 qualitative questionnaires containing open-ended questions about parents’ experiences of guilt and shame (Rúdólfsdóttir and Auðardóttir, 2024). Another study analyzed 63 self-written narratives, ranging from a few sentences to three pages, in which Finnish mothers described “forbidden” maternal emotions (Rotkirch and Janhunen, 2010). One study used in-depth case studies of different mothers to illustrate the manifestation of “separation guilt” (Baum, 2007).

With respect to study design, most of the reviewed studies employed cross-sectional qualitative designs. Only a couple adopted a longitudinal approach, following mothers over several months (Pescud and Pettigrew, 2014) and over a longer period, from pregnancy through the child’s first year of life (Holcomb, 2017).

Compared with quantitative research, qualitative studies encompass maternal guilt across a broader range of children’s developmental stages. Several of the included studies specifically examined maternal guilt among mothers of older children (up to 21 years of age), addressing less commonly discussed sources of guilt, such as children’s poor academic performance (Dudley-Marling, 2001), self-blame and guilt related to children’s and adolescents’ experiences of sexual abuse and health problems (Jackson and Mannix, 2004), and adolescents’ self-harm attempts (McDonald et al., 2007). However, in nearly half of the qualitative studies reviewed, children’s age was not a central consideration and was either not reported or not emphasized.

The recruitment of participants in the qualitative studies considered here was done predominantly through snowball sampling, starting from authors’ social networks (Elvin-Nowak, 1999; Aarntzen et al., 2019; Collins, 2021; Li, 2023) but also through purposive sampling, that involves selecting subjects relevant for the study (e.g., mothers who resigned after childbirth; Manna et al., 2021). Other modalities included placing study announcements in designated areas (Seagram and Daniluk, 2002), e-mail communications (Jackson and Mannix, 2004), interview invitations sent to mothers (and to their partners) who were previously part of a survey and gave their consent for future involvement in research (Calarco et al., 2021), accessing respondents through local institutions (Fielding-Singh and Cooper, 2023), or social media posts (Montano et al., 2023).

The reviewed qualitative studies investigated a diverse range of subgroups. Beyond the broad category of “working mothers,” several studies focused on professional groups exposed to unique work-related pressures, such as nurses (Öke Karakaya et al., 2021) and teachers (Guendouzi, 2006). Highly educated mothers were also the focus of several studies (Rubin and Wooten, 2007; Hillier, 2020; Fielding-Singh and Cooper, 2023). In addition, studies examining the influence of cultural identity on maternal guilt focused on migrant mothers in China (Li, 2022, 2023), Black mothers (Miller-Roenigk et al., 2026), or Latina mothers (Montano et al., 2023). Other studies explored maternal guilt in the context of economic difficulties and employment transitions (Pescud and Pettigrew, 2014; Manna et al., 2021). Finally, several studies examined mothers facing specific parenting challenges, including raising children with “school trouble” (Dudley-Marling, 2001), parenting adolescents who engaged in self-harm (McDonald et al., 2007), and experiencing intrusive or “bad thoughts” (Murray and Finn, 2012). These studies capture unique perspectives on maternal guilt (Table 3).

**Table 3:** Research relying on mixed methods.

No.	Author(s)	Country	Sample size	Children’s age	Measurement of guilt
1	Aarntzen et al. (2019)	Netherlands	Study 1 (qualitative): 14 dual-earner couples (28 parents); Study 2 (quantitative): 122 working mothers	Study 1: At least 1 child under 6 years; Study 2: Children under 13 years	Study 1: Semi-structured face-to-face interviews; Study 2: 8-day daily diary; single-item 5-point Likert scale (“When you think about how you combined work and family today.”).

## Sources of maternal guilt

Four main sources of guilt were identified across the 52 studies included in this review: maternal responsibility, identity conflict, interaction with children, and idealization of the maternal role. These categories were derived from an analysis of the theoretical perspectives and variables used in the studies, each capturing a dominant underlying trigger of guilt. Maternal responsibility concerns perceived failures to fulfil caregiving obligations and meet self-imposed standards regarding how caregiving is allocated and performed. Identity conflict captures tensions between motherhood and other personal or professional roles. Interaction with children concerns the perceived inadequacy of the quality or quantity of mother–child interactions, as well as children’s verbal criticism and emotionally distant behavior. Finally, idealization of the maternal role captures guilt arising from unattainable cultural standards of the “good mother” and broader social expectations surrounding motherhood. Organizing the reviewed studies around these four categories helps disentangle the underlying sources of maternal guilt across a diverse body of qualitative and quantitative research, while highlighting its multidimensional nature.

### Maternal responsibility

A primary driver of maternal guilt is the “unrelenting and total sense of responsibility” mothers feel for their children’s health, well-being, and development (Seagram and Daniluk, 2002, p. 66). Studies show that mothers feel frequently responsible for any aspect of children’s lives, from not spending enough time with or giving enough attention to children (Hillier, 2020), to broader aspects of children’s wellbeing and development, including harms (Seagram and Daniluk, 2002) and self-harms (in the case of pre-adolescents) (McDonald et al., 2007). This sense of duty extends to (infant) feeding practices and food preparation, where mothers feel accountable in relation to their children’s nutrition and healthy development (Pescud and Pettigrew, 2014; Holcomb, 2017). Another area in which mothers experience feelings of responsibility concerns their children’s academic performance (Dudley-Marling, 2001). Seeking support with childcare can also trigger strong maternal guilt (Calarco et al., 2021; Kerr et al., 2021), as it can be perceived as falling short of one’s maternal responsibilities. Additionally, maternal guilt related to perceived responsibility can be intensified by external blame and judgment (Jackson and Mannix, 2004).

### Identity conflict

Motherhood acts as a fundamental mandate of female identity, often leading to internal conflict when it clashes with mothers’ separate roles and associated needs and commitments. This domain highlights mothers’ constant habit of making choices and compromises. Research identifies that working long hours and experiencing challenges in balancing family and work responsibilities are significant sources of guilt for mothers (Borelli et al., 2016; Aarntzen et al., 2019, 2023). Becoming a mother can also be understood as a form of identity conflict, as women are often required to prioritize maternal duties over their own needs. This adjustment is especially demanding in the early stages of motherhood, when new mothers must transition into unfamiliar responsibilities, often in an overwhelming way (Murray and Finn, 2012; Dunford and Granger, 2017; Law et al., 2021). Engaging in self-care practices, such as hobbies, presents another conflict, requiring mothers to adopt alternative narratives to justify prioritizing a recreational activity over childcare (e.g., engaging in self-compassion; Miller and Strachan, 2020). The guilt felt by mothers initiating divorce, previously conceptualized as “separation guilt” (Baum, 2007), also underlines the conflict between a mothers’ desire to maintain the unity of the family and the need to exit a dysfunctional relationship.

### Interaction with children

The mother–child relationship is the central context in which maternal guilt arises. Children’s responses and behaviors significantly influence mothers’ feelings of guilt (Derella and Milan, 2021; Li, 2023). The way in which guilt is moderated in relation to the child itself was largely overlooked in previous research, but there are studies showing that children’s feedback is important in specific situations. For instance, in investigating Chinese migrant working mothers’ guilt, Li (2023) talked about the relational facet of maternal guilt, observing that children may have an active role in intensifying or alleviating their mothers’ guilt, especially when mothers do not align with predominant mothering expectations, such as prioritizing childcare and spending time with children (i.e., spending long periods of time working away from home). Interactional guilt was analyzed also in the context of children’s manifestation of negative emotions and their effect on mothers’ wellbeing (Derella and Milan, 2021). To manage guilt arising from problems related to the quality and quantity of mother–child interaction, mothers often engage in “reparative” parenting, such as overindulgence, sacrificing personal me-time, or planning to reduce working hours to intensify time with their children (Aarntzen et al., 2019; Li, 2023).

### Idealization of motherhood

The “intensive mothering” ideology (Hays, 1996) and the “motherhood myth” (Rich, 2021) capture the unattainable standards that mothers are subjected to, that may lead to maternal feelings of inadequacy. Research finds that these idealized standards can make mothers feel that any deviation from the ideal is a personal failure rather than a social or cultural problem (Sullivan, 2015). Even when mothers recognize these standards are unrealistic, they often still use them as a benchmark for self-evaluation. This is particularly evident in maternal food work, where mothers not only strive to meet normative feeding ideals but constantly attempt to “do more” by raising the bar on their own caregiving standards (Fielding-Singh and Cooper, 2023; Karademir Hazır, 2024). Self-comparison, external negative evaluation, and blame significantly harm mothers’ well-being (Liss et al., 2013). While comparisons with motherhood ideals may occur at a more abstract, perceptual level, in some communities, violating cultural norms is felt more acutely due to direct influence of other women in the family. For example, Montano et al. (2023) illustrate this dynamic in their analysis of maternal guilt among Latina mothers, finding that working mothers experienced guilt for not being constantly present, along with a sense of inadequacy compared to other women in the family, such as grandmothers, who had been full-time mothers. Moreover, research has found that the idealization of motherhood shaped by social class differences can also contribute to maternal guilt (Li, 2022; Karademir Hazır, 2024). For instance, Li’s (2022) study of rural-to-urban migrant mothers in China shows how these mothers internalize urban middle-class parenting ideals, intensifying their feelings of guilt.

## Correlates of maternal guilt

Having discussed four main sources of maternal guilt, this section examines its correlates. We focus on correlates at the individual, situational, and societal levels. At the individual level, we consider personal characteristics associated with greater maternal guilt, focusing on age, education, and beliefs about women’s roles. At the situational level, we examine the role of specific favorable or unfavorable situations for maternal guilt, including partner support, access to domestic and childcare assistance, workplace support, and household financial conditions. Finally, at the societal level, we explore how broader institutional structures, such as public policies and cultural norms, are associated with both the experience and expression of maternal guilt.

### Individual level factors

Age relates to how maternal guilt is experienced. Younger mothers were found to be more sensitive to criticism and negative evaluation than older mothers (Kenny and Robinson, 2026). In addition, in the case of younger mothers, motherhood may occur during other important life transitions, such as being a student or establishing a career, making the experience even more demanding (Maclean et al., 2021; Bayyar et al., 2026). In the context of “parental media guilt,” younger age predicts higher levels of guilt regarding both a child’s screen time and the mother’s own phone use (Coyne et al., 2025). Younger mothers also report higher levels of work-interfering-with-family guilt (Maclean et al., 2021). This reflects younger mothers’ more limited work experience and recent transition to motherhood, which can intensify work–family conflict (Bayyar et al., 2026).

Education is another important individual characteristic associated with maternal guilt. Higher education is frequently associated with increased maternal guilt, often because it correlates with higher socioeconomic status and the adoption of more demanding parenting standards (Dunford and Granger, 2017; Aarntzen et al., 2021; Fielding-Singh and Cooper, 2023; Coyne et al., 2025). For instance, university-educated mothers often set higher standards for themselves in what concerns children’s nutrition and cognitive stimulation (Fielding-Singh and Cooper, 2023). By contrast, mothers with lower levels of education are more likely to experience guilt related to financial constraints, which result in difficulties in providing adequate nutrition (Pescud and Pettigrew, 2014) and support regarding children’s academic performance (Li, 2023). Looking at the connection between education and maternal guilt suggests that it is not a matter of which mothers feel more guilt (higher educated or lower educated), but rather of how education shapes the reasons mothers experience maternal guilt.

Beyond relatively stable individual characteristics such as age and education, mothers’ beliefs about women’s roles in the family also relate to their experience of maternal guilt. Research indicates that when mothers internalize traditional gender roles, which position women as primary caregivers and men as breadwinners, they are more susceptible to experiencing intense guilt when their actions deviate from these ideals (Aarntzen et al., 2023). For example, mothers who feel less entitled to employment than fathers report greater guilt about working outside the home (Elvin-Nowak, 1999; Borelli et al., 2016, 2017), which tends to increase when mothers work longer hours (Borelli et al., 2017).

### Situational level factors

Several situational or circumstantial factors relate to maternal guilt. Among the most important are support structures, including support at home from partners and paid or informal caregivers (e.g., grandparents), workplace support, and the household’s economic situation.

Partners likely play a key role in alleviating maternal guilt. A lack of instrumental and emotional support from a partner, such as failing to share child-related duties or providing empathetic understanding, has been found to contribute to work–family conflict and maternal guilt (Uysal Irak et al., 2020; Foucreault et al., 2023).

The type and availability of domestic support also matter. Employed mothers who receive voluntary help with domestic tasks report higher levels of guilt than those who use paid assistance (Uysal Irak et al., 2020). Researchers suggested that paid support might be perceived as higher quality and avoids the specific guilt associated with asking family for favors (Uysal Irak et al., 2020). In line with these findings, in the US, some affluent mothers use their income to buy “a clearer conscience” by hiring private caregivers (Collins, 2021, p. 22). This allows them to focus on work while ensuring their home remains a stable environment, thereby reinforcing their perception of themselves as “good mothers” (Collins, 2021). Mothers often feel guilty for asking their own parents or parents-in-law for assistance, viewing it as a failure to handle their own responsibilities (Rúdólfsdóttir and Auðardóttir, 2024).

The workplace relates to maternal guilt through immediate, concrete job demands and daily events that impede a mother’s ability to meet her caregiving responsibilities (Borelli et al., 2017; Aarntzen et al., 2021). Lack of understanding on behalf of employers is important in this context. Research shows that mothers who feel emotionally supported by their employers report lower levels of guilt (Uysal Irak et al., 2020) whereas unsupportive work environments may intensify feelings of conflict between work and family responsibilities (Manna et al., 2021). Broader organizational norms are also relevant. In some organizational contexts, mothers in the private sector report higher guilt due to more rigid hours and higher work demands compared to those in the public sector (Bayyar et al., 2026).

Finally, household financial circumstances also relate to maternal guilt. The financial strain is mediated through parenting practices about food and caregiving, which can trigger mothers’ feelings of guilt (Pescud and Pettigrew, 2014). Among single mothers, the financial burden and associated social stigma of raising a child in a low-income household are significant contributors to parenting guilt (Jackson and Mannix, 2004; Kim et al., 2023).

### Societal level factors

The institutional and cultural context significantly influences maternal guilt through national policies, healthcare standards, and educational expectations against which mothers are judged. Rather than being a purely individual psychological issue, research shows that maternal guilt is politically and culturally derived from how society structures and assigns caregiving responsibilities (Collins, 2021).

National work–family policies determine the extent to which childcare is treated as a private or public responsibility. Evidence from contrasting family policy regimes in the United States and Nordic countries, such as Iceland and Sweden, suggests that maternal guilt can be intensified both when institutional support is limited and when generous support is distributed unevenly between mothers and fathers. In the US, the lack of paid maternity leave, universal childcare, and structural support creates a “neoliberal logic” where childrearing becomes a private burden (Collins, 2021, p. 4). Paired with prevailing intensive mothering ideals (Hays, 1996), mothers feel primarily accountable for balancing work and caregiving responsibilities, which intensifies guilt (Collins, 2021). In contrast, in countries such as Sweden and Iceland, generous parental leave and high-quality, subsidized daycare are institutional norms (Collins, 2021; Rúdólfsdóttir and Auðardóttir, 2024). Yet, these benefits can also back-fire, as they enhance the expectation of mothers as caregivers, who may experience guilt if they are willing to return to work earlier (Rúdólfsdóttir and Auðardóttir, 2024).

The school system often reinforces the cultural standard that mothers are personally responsible for children’s academic results and conduct. When children struggle in school, mothers (rather than fathers) are typically expected to bear the associated emotional and logistical burden (Dudley-Marling, 2001).

Healthcare institutions often establish “best practice” recommendations that become cultural benchmarks for good mothering. For example, the promotion of breastfeeding is frequently framed as a moral choice, creating conditions in which mothers may experience guilt if they are unable or choose not to breastfeed (Williams et al., 2013). Moreover, medical advice encouraging mothers to “not feel guilty” about their feeding choices may offer only a superficial solution, as it fails to address the underlying cultural pressures that give rise to maternal guilt (Williams et al., 2013). The pressure to conform to medical standards extends throughout mothers’ parenting experience. Mothers are often held responsible for their children’s health-related issues, whereas fathers are largely exempt from such scrutiny (Jackson and Mannix, 2004). Research shows that healthcare providers frequently attribute children’s health problems to mothers, even when these are beyond their control, reflecting broader cultural expectations within healthcare systems (Jackson and Mannix, 2004).

At the societal level, religion acts as a powerful institutional force that reinforces motherhood as a sacred responsibility, often legitimizing traditional gender roles and providing a moral foundation for intensive mothering practices. Although religion received relatively limited attention in the reviewed studies, its influence is evident in several cultural contexts. For example, in India and Turkey, religion contributes to framing motherhood as a sacred role (Öke Karakaya et al., 2021; Siddiqi et al., 2025). Similarly, in Italy, cultural beliefs informed by Catholicism reinforce traditional gender norms in which men are expected to engage in paid work while women assume primary responsibility for the home and caregiving (Collins, 2021).

## Maternal guilt in cross-national research

Maternal guilt is a common emotion among mothers across the globe. However, some differences are observed across contexts. In regions with lower levels of gender equality, maternal guilt most commonly arises as mothers struggle to balance work and motherhood under constraining circumstances (Borelli et al., 2016, 2017; Manna et al., 2021; Öke Karakaya et al., 2021). Maternal guilt continues to arise from the tension between work and motherhood also in regions with higher levels of gender equality, but it is more closely associated with making the right decisions for children, being physically and emotionally present, and striving to meet ideals of “perfect” parenting (Rotkirch and Janhunen, 2010; Rúdólfsdóttir and Auðardóttir, 2024).

Cross-national comparative studies regarding maternal guilt remain scarce and provide somewhat contradictory evidence (Collins, 2021; Aarntzen et al., 2023). Nevertheless, this body of research is essential for understanding how different national contexts shape mothers’ experiences of guilt. For example, Collins (2021) compared maternal guilt in Sweden, Germany, Italy, and the US, focusing on the role of welfare regimes and public policies. The study finds that in Sweden, mothers benefit from generous parental leave but are also expected to participate in the labor market. In Germany, social policies encourage a model of extended maternity leave followed by part-time work, contributing to greater guilt among mothers who choose to work full-time. By contrast, in Italy and the US, limited state support intensifies maternal guilt by framing childrearing primarily as a private responsibility (Collins, 2021). Aarntzen et al. (2021) present another comparative cross-national study on a specific form of guilt: work-family guilt. Relying on European Sustainable Workforce Survey, nine countries are included and compared: Bulgaria, Finland, Germany, Hungary, Portugal, Spain, Sweden, the Netherlands, and the United Kingdom. The countries, however, are compared based on national gender norms disregarding the institutional complexity of each country. Aarntzen et al. (2021) do not find evidence that national gender norms explain differences across countries in parents’ work–family guilt. Regardless of a country’s profile, work–family guilt is primarily shaped by parents’ individual beliefs and their immediate organizational environment. Taking a different approach, Siddiqi et al. (2025) compared maternal employment-related guilt in India and the US but focused on its outcomes rather than its associated factors.

Overall, cross-national research does not reach a consensus on the country differences in how maternal guilt is experienced, indicating both that maternal guilt is embedded within national contexts and that the context might be less relevant when individual beliefs and situational and organizational environments are considered.

## Summary, discussion and future research directions

We conclude our narrative review of maternal guilt by summarizing and discussing the main findings and suggesting directions for future research. Drawing on 52 studies across different disciplines, the review has examined the theoretical foundations and measurement of maternal guilt, synthesized its sources and correlates, and assessed the limited cross-national evidence. While interdisciplinary in scope, the review emphasizes a sociological perspective on maternal guilt.

Two theoretical perspectives inform sociological explanations of maternal guilt: the dramaturgical perspective highlights negative emotions arising when mothers’ experiences conflict with socially established feeling rules, while symbolic interactionism emphasizes tensions between motherhood and other identities. Together, these perspectives illuminate distinct but complementary mechanisms through which maternal guilt is socially constructed.

The review identifies four main sources of maternal guilt: maternal responsibility, identity conflict, interaction with children, and idealization of the maternal role. These categories are not mutually exclusive but capture different underlying triggers of guilt. Maternal responsibility concerns perceived failures to meet caregiving obligations, while interaction with children concerns difficulties in mother–child relationships and the perceived inadequacy of time and attention devoted to children. Identity conflict captures tensions between motherhood and other identities, while idealization of the maternal role concerns perceived failures to meet cultural standards of the “good mother.”

We examine maternal guilt as a social phenomenon and a socially shaped emotion associated with factors at multiple levels. These include individual characteristics such as age, education, and social beliefs; situational conditions such as family context, domestic support, financial circumstances, and workplace characteristics; and societal factors such as work–family policies, healthcare institutions, and religious beliefs. Maternal guilt should be thus understood in relation to individual circumstances and broader structural expectations.

The overview of the field shows a number of significant contributions that measure and analyze maternal guilt, spanning from quantitative and psychometric studies to qualitative research. While this heterogeneity represents an important aspect of the evidence base, it may influence not only the findings reported in individual studies but also the extent to which these findings can be meaningfully compared, synthesized, and generalized across contexts. In other words, findings that appear consistent across different settings may reflect genuinely shared patterns, but they may also result from differences in conceptual definitions or operationalization. Conversely, divergent findings should not necessarily be interpreted as contradictory, as they may be partly explained by contextual specificities or methodological choices. Related to this, critical gaps concern cross-national variation. Existing research has so far focused mostly on individual countries, with only few comparative studies (Aarntzen et al., 2021; Collins, 2021). Research shows that maternal guilt persists even in egalitarian settings, such as Sweden (Collins, 2021) and Finland (Rotkirch and Janhunen, 2010). However, these findings cannot directly be compared with those from other countries. Comparative research across a wider range of countries would be an important step forward. Several other directions for future research also emerge from this review.

First, more research is needed on differences in maternal guilt across population subgroups. While most qualitative and quantitative studies have focused on the experiences of middle-class, educated mothers, financial struggles and their role in maternal guilt remain underexplored (e.g., Pescud and Pettigrew, 2014; Kim et al., 2023). More insights are needed for groups such as single mothers, migrant mothers, adolescent mothers, mothers with disabilities, sexual minority mothers, incarcerated mothers or mothers with unique responsibilities would help tailor interventions to address their specific needs.

The comparison with fathers is also largely missing. Most of the reviewed qualitative studies have focused on interviewing mothers, offering an in-depth understanding of their experiences. In contrast, quantitative studies have included some comparisons with fathers, leading to a broader but less nuanced exploration of maternal (and paternal) guilt. Therefore, future research should seek to examine various manifestations of paternal guilt, comparing its intensity and types to maternal guilt.

Digitalization is a recent phenomenon, and research on maternal guilt related to the use of digital devices by children and adolescences is currently limited (e.g., Coyne et al., 2025). The role of digital environments in maternal guilt is an emerging area to consider. Researchers could investigate how the use of digital devices among children contributes to maternal guilt, particularly when mothers feel pressured to comply with peer practices regarding technology use. Moreover, as the use of social platforms has become a normality, future research could investigate how the use of social media influences maternal guilt. This is closely related to the need to clarify which parental challenges are specific to different stages of children’s development and how they contribute to maternal guilt.

Lastly, methodological approaches to maternal guilt can also be further developed. Longitudinal and experimental research, which remains limited, could provide valuable insights into how maternal guilt evolves over time and how policy and organizational interventions shape its development.

Importantly, the current literature mostly deals with present emotions and there is the absence of analyses of anticipated maternal guilt. Because of social norms and the experiences of their family and friends, childless women may anticipate feelings of maternal guilt that could influence their future behavior. Future studies could explore how childless women envision maternal guilt and whether these anticipations of guilt, as a product of internalized and societal expectations, influence their life.

Ultimately, this review shows that understanding maternal guilt requires attention to how the social organization of motherhood shapes both the expectations placed on mothers and their opportunities to meet them, situating their emotional experiences within the relationships, institutions, and cultural norms that structure their lives.

## References

[ref1] Aarntzen L. Derks B. van Steenbergen E. Ryan M. van der Lippe T. (2019). Work-family guilt as a straightjacket. An interview and diary study on consequences of mothers’ work-family guilt. J. Vocat. Behav. 115:103336. doi: 10.1016/j.jvb.2019.103336

[ref2] Aarntzen L. van der Lippe T. van Steenbergen E. Derks B. (2021). How individual gender role beliefs, organizational gender norms, and national gender norms predict parents’ work-family guilt in Europe. Community Work Fam. 24, 120–142. doi: 10.1080/13668803.2020.1816901

[ref3] Aarntzen L. Derks B. van Steenbergen E. van der Lippe T. (2023). When work–family guilt becomes a women’s issue: internalized gender stereotypes predict high guilt in working mothers but low guilt in working fathers. Br. J. Soc. Psychol. 62, 12–29. doi: 10.1111/bjso.12575, 36097879 PMC10087844

[ref4] Acker J. (1990). Hierarchies, jobs, bodies: a theory of gendered organizations. Gend. Soc. 4, 139–158. doi: 10.1177/089124390004002002

[ref5] Aycan Z. Eskin M. (2005). Relative contributions of childcare, spousal support, and organizational support in reducing work-family conflict for men and women: the case of Turkey. Sex Roles 53, 453–471. doi: 10.1007/s11199-005-7134-8

[ref6] Batram-Zantvoort S. Wandschneider L. Niehues V. Razum O. Miani C. (2022). Maternal self-conception and mental wellbeing during the first wave of the COVID-19 pandemic. A qualitative interview study through the lens of “intensive mothering” and “ideal worker” ideology. Front. Glob. Women's Health 3:1–16 doi: 10.3389/fgwh.2022.878723, 36132187 PMC9484320

[ref7] Baum N. (2007). “Separation guilt” in women who initiate divorce. Clin. Soc. Work. J. 35, 47–55. doi: 10.1007/s10615-006-0053-5

[ref8] Bayyar A. B. Daşbaş S. Kıraç R. (2026). Investigating the relationship between maternal employment guilt, perceived social support, and job satisfaction. J. Fam. Issues 1–26. doi: 10.1177/0192513X261423881

[ref9] Benoit B. Goldberg L. Campbell-Yeo M. (2016). Infant feeding and maternal guilt: the application of a feminist phenomenological framework to guide clinician practices in breast feeding promotion. Midwifery 34, 58–65. doi: 10.1016/j.midw.2015.10.011, 26612001

[ref10] Borelli J. L. Nelson S. K. River L. M. Birken S. A. Moss-Racusin C. (2016). Gender differences in work-family guilt in parents of young children. Sex Roles 76, 356–368. doi: 10.1007/s11199-016-0579-0

[ref11] Borelli J. L. Nelson-Coffey S. K. River L. M. Birken S. A. Moss-Racusin C. (2017). Bringing work home: gender and parenting correlates of work-family guilt among parents of toddlers. J. Child Fam. Stud. 26, 1734–1745. doi: 10.1007/s10826-017-0693-9

[ref12] Calarco J. M. Meanwell E. Anderson E. M. Knopf A. S. (2021). By default: how mothers in different-sex dual-earner couples account for inequalities in pandemic parenting. Socius 7:1–15. doi: 10.1177/23780231211038783

[ref13] Collins C. (2021). Is maternal guilt a cross-national experience? Qual. Sociol. 44, 1–29. doi: 10.1007/s11133-020-09451-2

[ref14] Constantinou G. Varela S. Buckby B. (2021). Reviewing the experiences of maternal guilt–the “motherhood myth” influence. Health Care Women Int. 42, 852–876. doi: 10.1080/07399332.2020.1835917, 33600296

[ref15] Coyne S. M. Hirschi T. Gale M. Jones B. L. Van Alfen M. Almeida L. M. . (2025). “I’ve let screens take over”: maternal guilt about media and relations to children’s problematic media use [Preprint]. SSRN Electron. J. doi: 10.2139/ssrn.5529557 PMC13492524 42626652

[ref16] Derella O. J. Milan S. (2021). I felt like a terrible mom: parenting-related cognitive processes maintaining maternal depression. J. Child Fam. Stud. 30, 2427–2439. doi: 10.1007/s10826-021-02053-8

[ref17] Dudley-Marling C. (2001). School trouble: a mother’s burden. Gender Educ. 13, 183–197. doi: 10.1080/09540250120051196

[ref18] Dunford E. Granger C. (2017). Maternal guilt and shame: relationship to postnatal depression and attitudes towards help-seeking. J. Child Fam. Stud. 26, 1692–1701. doi: 10.1007/s10826-017-0690-z

[ref19] Elvin-Nowak Y. (1999). The meaning of guilt: a phenomenological description of employed mothers’ experiences of guilt. Scand. J. Psychol. 40, 73–83. doi: 10.1111/1467-9450.00100, 10216466

[ref20] Fielding-Singh P. Cooper M. (2023). The emotional management of motherhood: foodwork, maternal guilt, and emotion work. J. Marriage Fam. 85, 436–457. doi: 10.1111/jomf.12878

[ref21] Foucreault A. Ménard J. Houlfort N. Trépanier S.-G. Lavigne G. L. (2023). How work-family guilt, involvement with children and spouse’s support influence parents’ life satisfaction in a context of work-family conflict. J. Child Fam. Stud. 32, 1065–1077. doi: 10.1007/s10826-022-02443-6, 36186677 PMC9512965

[ref22] Gonçalves G. Sousa C. Santos J. Silva T. Korabik K. (2018). Portuguese mothers and fathers share similar levels of work-family guilt according to a newly validated measure. Sex Roles 78, 194–207. doi: 10.1007/s11199-017-0782-7, 30311153

[ref23] Guendouzi J. (2006). “The guilt thing”: balancing domestic and professional roles. J. Marriage Fam. 68, 901–909. doi: 10.1111/j.1741-3737.2006.00303.x

[ref24] Haslam D. Filus A. Finch J. (2020). The guilt about parenting scale (GAPS): development and initial validation of a self-report measure of parenting guilt, and the relationship between parenting guilt and work and family variables. J. Child Fam. Stud. 29, 880–894. doi: 10.1007/s10826-019-01565-8

[ref25] Hays S. (1996). The Cultural Contradictions of Motherhood. New Haven, CT: Yale University Press.

[ref26] Hillier K. M. (2020). Experiences of maternal guilt and intensive mothering ideologies in graduate student mothers, faculty, and sessional instructors. J. Mother Stud. 5. https://jourms.org/experiences-of-maternal-guilt-and-intensive-mothering-ideologies-in-graduate-student-mothers-faculty-and-sessional-instructors/2026

[ref27] Hochschild A. R. (1979). Emotion work, feeling rules, and social structure. Am. J. Sociol. 85, 551–575. doi: 10.1086/227049

[ref28] Holcomb J. (2017). Resisting guilt: mothers’ breastfeeding intentions and formula use. Sociol. Focus 50, 361–374. doi: 10.1080/00380237.2017.1313005

[ref29] Jackson D. Mannix J. (2004). Giving voice to the burden of blame: a feminist study of mothers’ experiences of mother blaming. Int. J. Nurs. Pract. 10, 150–158. doi: 10.1111/j.1440-172X.2004.00474.x, 15265225

[ref30] Jackson L. de Pascalis L. Harrold J. Fallon V. (2021). Guilt, shame, and postpartum infant feeding outcomes: a systematic review. Matern. Child Nutr. 17:e13141. doi: 10.1111/mcn.13141, 33491303 PMC8189225

[ref31] Jackson L. Fallon V. Harrold J. De Pascalis L. (2022). Maternal guilt and shame in the postpartum infant feeding context: a concept analysis. Midwifery 105:103205. doi: 10.1016/j.midw.2021.103205 34864327

[ref32] Karademir Hazır I. (2024). Guilt and beyond: a class cultural analysis of evolving emotional responses to maternal foodwork. Sociology 58, 1399–1415. doi: 10.1177/00380385241244994

[ref33] Kenny M. Robinson O. C. (2026). Investigating relationships between guilt, shame, self-discrepancy and fear of negative evaluation in mothers: a replication and extension. J. Child Fam. Stud. 35, 1292–1303. doi: 10.1007/s10826-026-03274-5

[ref34] Kerr M. L. Rasmussen H. F. Smiley P. A. Fanning K. A. Buttitta K. V. Benson L. . (2021). Within- and between-family differences in mothers’ guilt and shame: caregiving, coparenting, and attachment. J. Fam. Psychol. 35:265–275. doi: 10.1037/fam0000647

[ref35] Kim A. Jeon S. Park I. (2023). Influence of parenting guilt on the mental health among single-parent women: multiple additive moderating effect of economic well-being and level of education. Healthcare 11:1–12. doi: 10.3390/healthcare11131814, 37444650 PMC10340295

[ref36] Korabik K. (2015). “The intersection of gender and work–family guilt,” in Gender and the Work-Family Experience, ed. Mills M. (Cham: Springer). doi: 10.1007/978-3-319-08891-4_8

[ref37] Law N. K. Hall P. L. Cheshire A. (2021). Common negative thoughts in early motherhood and their relationship to guilt, shame and depression. J. Child Fam. Stud. 30, 1831–1845. doi: 10.1007/s10826-021-01968-6

[ref38] Li M. (2022). “Only mother is the best in the world”: maternal guilt, migrant motherhood, and changing ideologies of childrearing in China. J. Fam. Commun. 22, 87–103. doi: 10.1080/15267431.2021.2019742

[ref39] Li M. (2023). Guilt and compensation: the interplay between maternal emotions and parent–child relationships in migrant families. Fam. Relat. 72, 1607–1624. doi: 10.1111/fare.12784

[ref40] Liss M. Schiffrin H. H. Rizzo K. M. (2013). Maternal guilt and shame: the role of self-discrepancy and fear of negative evaluation. J. Child Fam. Stud. 22, 1112–1119. doi: 10.1007/s10826-012-9673-2

[ref41] Maclean E. I. Andrew B. Eivers A. (2021). The motherload: predicting experiences of work-interfering-with-family guilt in working mothers. J. Child Fam. Stud. 30, 169–181. doi: 10.1007/s10826-020-01852-9

[ref42] Mann M. B. Thornburg K. R. (1987). Guilt of working women with infants and toddlers in day care. Early Child Dev. Care 27, 451–464. doi: 10.1080/0300443870270304

[ref43] Manna V. Procentese F. Di Napoli I. Arcidiacono C. (2021). Helpless mothers dropping out of the workplace: the Italian case of voluntary resignation. Qual. Rep. 26, 1179–1199. doi: 10.46743/2160-3715/2021.4490

[ref44] Marschall D. Sanftner J. Tangney J. (1994). The state shame and Guilt scale. VA: George Mason University Fairfax.

[ref45] Martínez P. Carrasco M. J. Aza G. Blanco A. Espinar I. (2011). Family gender role and guilt in Spanish dual-earner families. Sex Roles 65, 813–826. doi: 10.1007/s11199-011-0031-4

[ref46] McDonald G. O’Brien L. Jackson D. (2007). Guilt and shame: experiences of parents of self-harming adolescents. J. Child Health Care 11, 298–310. doi: 10.1177/1367493507082759, 18039732

[ref47] McElwain A. K. (2008). “Doctoral dissertation,” in An Examination of the Reliability and Validity of the Work-Family Guilt Scale, (University of Guelph). Ontario, Canada.

[ref48] Miller C. L. Strachan S. M. (2020). Understanding the role of mother guilt and self-compassion in health behaviors in mothers with young children. Women Health 60, 763–775. doi: 10.1080/03630242.2020.1713966, 31937201

[ref49] Miller-Roenigk B. D. Thorpe S. Palomino K. A. Stevens-Watkins D. (2026). Breastfeeding barriers among black mothers: maternal guilt, shame, and other affective stressors. Women Ther. 49, 195–222. doi: 10.1080/02703149.2026.2633905

[ref50] Montano M. Mizock L. Pulido C. Calzada E. (2023). The maternal guilt of working Latina mothers: a qualitative study. Hisp. J. Behav. Sci. 45, 149–171. doi: 10.1177/07399863241239991

[ref51] Murray L. Finn M. (2012). Good mothers, bad thoughts: new mothers’ thoughts of intentionally harming their newborns. Feminism Psychol. 22, 41–59. doi: 10.1177/0959353511414015

[ref52] Öke Karakaya P. Sönmez S. Aşık E. (2021). A phenomenological study of nurses’ experiences with maternal guilt in Turkey. J. Nurs. Manag. 29, 2515–2522. doi: 10.1111/jonm.13401, 34216495

[ref53] Orth U. Berking M. Burkhardt S. (2006). Self-conscious emotions and depression: rumination explains why shame but not guilt is maladaptive. Pers. Soc. Psychol. Bull. 32, 1608–1619. doi: 10.1177/0146167206292958, 17122174

[ref54] Pescud M. Pettigrew S. (2014). “I know it’s wrong, but”: a qualitative investigation of low-income parents’ feelings of guilt about their child-feeding practices. Matern. Child Nutr. 10:422–435. doi: 10.1111/j.1740-8709.2012.00425.x, 22708589 PMC6860221

[ref55] Rich A. (2021). Of Woman Born: Motherhood as Experience and Institution. New York: W. W. Norton & Company.

[ref56] Rotkirch A. Janhunen K. (2010). Maternal guilt. Evol. Psychol. 8, 90–106. doi: 10.1177/147470491000800108, 22947781 PMC10480956

[ref57] Rubin S. E. Wooten H. R. (2007). Highly educated stay-at-home mothers: a study of commitment and conflict. Fam. J. 15, 336–345. doi: 10.1177/1066480707304945

[ref58] Rúdólfsdóttir A. G. Auðardóttir A. M. (2024). “I feel like I am betraying my child”: the socio-politics of maternal guilt and shame. Gend. Work Organ. 31, 2733–2748. doi: 10.1111/gwao.13124, 40046247

[ref59] Seagram S. Daniluk J. C. (2002). “It goes with the territory”: the meaning and experience of maternal guilt for mothers of preadolescent children. Women Ther. 25, 61–88. doi: 10.1300/J015v25n01_04

[ref60] Shott S. (1979). Emotion and social life: a symbolic interactionist. Am. J. Sociol. 84, 1317–1334. doi: 10.1086/226936

[ref61] Siddiqi N. Liss M. Ullah F. I. (2025). Exploring the effects of maternal employment guilt on the personal and professional well-being of working moms: a cross-cultural investigation. Gend. Issues 42:31. doi: 10.1007/s12147-025-09377-5

[ref62] Slobodin O. Cohen R. Arden A. Katz I. (2020). Mothers’ need frustration and controlling parenting: the moderating role of maternal guilt. J. Child Fam. Stud. 29, 1914–1926. doi: 10.1007/s10826-020-01720-6

[ref63] Song T. Pan C. Wang J. Li M. (2025). When women’s work benefits family: the impact of mothers’ work-family enrichment on parenting guilt. Health Care Women Int. 9, 1–17. doi: 10.1080/07399332.2025.2569579, 41066204

[ref64] Sullivan C. (2015). “Bad mum guilt”: the representation of “work-life balance” in UK women’s magazines. Community Work Fam. 18, 284–298. doi: 10.1080/13668803.2014.970128

[ref65] Sutherland J. (2010). Mothering, guilt and shame. Sociol. Compass 4, 310–321. doi: 10.1111/j.1751-9020.2010.00283.x

[ref66] Turner J. H. Stets J. E. (2006). Sociological theories of human emotions. Annu. Rev. Sociol. 32, 25–52. doi: 10.1146/annurev.soc.32.061604.123130

[ref67] Uysal Irak D. Kalkışım K. Yıldırım M. (2020). Emotional support makes the difference: work-family conflict and employment related guilt among employed mothers. Sex Roles 82, 53–65. doi: 10.1007/s11199-019-01035-x

[ref68] Williams K. Kurz T. Summers M. Crabb S. (2012). Discursive constructions of infant feeding: the dilemma of mothers’ “guilt”. Fem. Psychol. 23, 339–358. doi: 10.1177/0959353512444426

[ref69] Williams K. Donaghue N. Kurz T. (2013). “Giving guilt the flick”?: an investigation of mothers’ talk about guilt in relation to infant feeding. Psychol. Women Q. 37, 97–112. doi: 10.1177/0361684312463000

[ref70] Wolfers L. N. Wendt R. Becker D. Utz S. (2023). Do you love your phone more than your child? The consequences of norms and guilt around maternal smartphone use. Hum. Commun. Res. 49, 285–295. doi: 10.1093/hcr/hqad001

[ref71] Yüce-Selvi Ü. Kantaş Ö. (2019). The psychometric evaluation of the maternal employment guilt scale: a development and validation study. İş Güç J. Ind. Relat. Hum. Resour. 21, 27–52. doi: 10.4026/isguc.543449

